# Conference Report: Cerebellar Development and Disease at Single-Cell Resolution

**DOI:** 10.1007/s12311-025-01864-5

**Published:** 2025-06-05

**Authors:** Lena M. Kutscher, Davide Aprile, N. Sumru Bayin, Esther B. E. Becker, Valentina Cerrato, Giacomo Turrini, Marion Coolen, Vincent Cantagrel, Béatrice C. Durand, Myron K. Evans II, Parthiv Haldipur, Kathleen J. Millen, Joanna Yeung, Daniel Goldowitz, Mary E. Hatten, Alexandra L. Joyner, Justus M. Kebschull, James Y. H. Li, Giorgia Quadrato, Christin Schmidt, Mari Sepp, Teresa P. Silva, Giuseppe Testa, Luca Tiberi, Simone Mayer

**Affiliations:** 1 Developmental Origins of Pediatric Cancer Junior Research Group, Hopp Children’s Cancer Center (KiTZ) and German Cancer Research Center (DKFZ), Heidelberg, Germany; 2 Human Technopole, Viale Rita Levi-Montalcini 1, 20157 Milan, Italy; 3 Gurdon Institute, University of Cambridge, Cambridge, UK; 4 Department of Physiology, Development and Neuroscience, University of Cambridge, Cambridge, UK; 5 Nuffield Department of Clinical Neurosciences, University of Oxford, Oxford, UK; 6 Kavli Institute of Nanoscience Discovery, University of Oxford, Oxford, UK; 7 Department of Neuroscience Rita LeviMontalcini, University of Turin, Turin, Italy; 8 Neuroscience Institute Cavalieri Ottolenghi, Orbassano, Turin, Italy; 9 Developmental Brain Disorders Laboratory, Imagine Institute, INSERM UMR 1163, Université Paris Cité, Paris, France; 10 Sorbonne Université, CNRS UMR8263, Institut de Biologie Paris-Seine (IBPS) - “Développement, Adaptation et Vieillissement” (Dev2A), Paris, France; 11 Ben Towne Center for Childhood Cancer and Blood Disorders Research, Seattle Children’s Research Institute, Seattle, WA USA; 12 Department of Pediatrics, University of Washington School of Medicine, Seattle, WA USA; 13 Center for Integrative Brain Research, Seattle Children’s Research Institute, Seattle, WA USA; 14 Centre for Molecular Medicine and Therapeutics, British Columbia Children’s Hospital Research Institute, Vancouver, BC Canada; 15 Department of Medical Genetics, Centre for Molecular Medicine and Therapeutics, University of British Columbia, Vancouver, BC Canada; 16 Laboratory of Developmental Neurobiology, The Rockefeller University, New York, NY USA; 17 Developmental Biology Program, Memorial Sloan Kettering Cancer Center, New York, NY USA; 18 Department of Biomedical Engineering, Johns Hopkins University, Baltimore, MD USA; 19 Kavli Neuroscience Discovery Institute, Johns Hopkins University, Baltimore, MD USA; 20 Department of Genetics and Genome Sciences, University of Connecticut School of Medicine, Farmington, CT USA; 21 Institute for Systems Genomics, University of Connecticut, Farmington, CT USA; 22 Department of Stem Cell Biology and Regenerative Medicine, Keck School of Medicine, University of Southern California, Los Angeles, CA USA; 23 Eli and Edythe Broad CIRM Center for Regenerative Medicine and Stem Cell Research at USC, University of Southern California, Keck School of Medicine, Los Angeles, CA USA; 24 Department of Neurology, University of California San Francisco, San Francisco, CA USA; 25 Center for Molecular Biology of Heidelberg University (ZMBH), Heidelberg, Germany; 26 Developmental Biology and Cancer Department, UCL GOS Institute of Child Health, London, UK; 27 Department of Oncology and Hemato-Oncology, University of Milan, Milan, Italy; 28 Armenise-Harvard Laboratory of Brain Cancer, University of Trento, Trento, Italy; 29 Karlsruhe Institute of Technology, Zoological Institute, Karlsruhe, Germany; 30 Karlsruhe Institute of Technology, Institute of Biological and Chemical Systems - Functional Molecular Systems (IBCS-FMS), Karlsruhe, Germany

The application of single-cell omics has revolutionized how we understand the cellular and molecular mechanisms underlying development and disease, particularly in the brain, with its large number of distinct cell types and its high degree of regionalization. Previous research has largely focused on the neocortex, where single-cell transcriptomics have revealed cell type-specific gene expression patterns throughout development in many species and dysregulation of such expression patterns in various neurological disorders. While well-established conferences exist for neocortical development and disease, the cerebellar development research community lacked a unifying conference. Given the importance of the cerebellum in many brain functions including motor and cognitive functions, and given its role in widespread neurodevelopmental disorders, including autism spectrum disorder, ataxia, and cancer, as well as its involvement in rare genetic disorders such as pontocerebellar hypoplasia, a unifying cerebellar development conference was urgently needed.

On Sept 11-13th, 2024, 55 researchers from around the world (8 countries: Canada, France, Germany, Italy, Netherlands, Sweden, United Kingdom, United States of America) gathered in Heidelberg, Germany, for the first “Cerebellar development and disease at single-cell resolution” conference. 39 participants held a PhD, 16 were trainees. 60% of the participants were women. This conference was the first of its kind, dedicated to the genes, pathways and models of cerebellar development. We brought together new and established leaders in the field of cerebellar development for three days full of interactions and discussions. Fifteen invited speakers, three selected abstract talks, and 15 posters were presented. Each talk (about 30 min) was followed by a discussion session (about 15 min). The conference was hosted at the Heidelberg Academy of Sciences and Humanities in the Old Town of Heidelberg, Germany, where the intimate setting allowed for extensive formal and informal discussions and networking.

This conference paper summarizes the ongoing and published research presented by the speakers. We highlight the current state-of-the-art in the field regarding developmental mechanisms in different species, new human in vitro models, and their application to a wide range of disorders.

## Molecular Models of Cerebellar Development (M.E. Hatten)

The role of the cerebellum is rapidly changing from simple motor control to complex cognitive tasks. Mary Beth Hatten reported on experiments showing how the dynamics of chromatin modifications change during neuronal differentiation. Their recent studies show that dynamic changes in the chromatin landscape regulate gene expression during cerebellar neurogenesis, migration and circuit formation. Those studies demonstrate that H3K4me3 and H3K27me3 histone bivalency regulates the timing of granule cell differentiation and glial-guided migration [1]. In other studies, they have studied *Astn2*, a gene that functions in migration and membrane receptor recycling and that has been implicated in autism spectrum disorders (ASD) and language delay. In a mouse model of *Astn2*, their work shows that mice lacking *Astn2* have ASD-like behaviors, region-specific changes in dendritic spine density and altered cerebellar circuit properties [2]. Electrophysiological experiments indicate a reduced frequency of spontaneous excitatory postsynaptic currents (EPSCs), as well as increased amplitudes of both spontaneous EPSCs and inhibitory postsynaptic currents (IPSCs) in the *Astn2* KO animals, suggesting that pre- and postsynaptic components of synaptic transmission are altered. Finally, the Hatten group has recently developed a robust method to generate human pluripotent stem cell (hPSC)-derived Purkinje cells (PCs) and designed a 3D microfluidic culture system to mimic native brain lamination. They are using the system to study circuit properties of human cerebellar neurons and to identify changes in molecular pathways in cerebellar disease models.

## Functional Implications of Molecular Subclasses of Cerebellar Excitatory Neurons (A.L. Joyner)

The excitatory neurons of the cerebellum include the granule cells, unipolar brush cells and those of the three cerebellar nuclei (CN) that constitute the primary output for the cerebellum. The excitatory neurons of the medial CN (eCNm) were recently divided into subdomains in the adult based on transcriptomics and circuit organization [3, 4]. However, how the subdomains are established during development is unclear. The Joyner group previously showed that mice lacking the homeobox genes *En1* and *En2* in all eCN (*Atoh1-En1/2* conditional knockouts (CKOs)) have preferential death of embryonic eCN in the medial and intermediate CN [5]. Using scRNA-seq and spatial expression analysis, they found the medial eCN are transcriptionally divergent from the other nuclei as early as E14.5 and identified molecular subdomains at this stage that evolve during embryogenesis to prefigure the adult [6]. Furthermore, *Atoh1-En1/2* CKOs undergo death of specific posterior eCNm molecular sub-domains. There also is a reduction in synaptic gene expression in mutant eCNm and TBR2 (EOMES) is down regulated in an anterior subdomain. Interestingly, the functions of EN1/2 in mediating TBR2 expression, neuron differentiation and survival are conserved in the other two excitatory neuron types. Using behavior assays, the Joyner group found *Atoh1-En1/2* CKOs have deficits in motor coordination during development and in adult motor learning, acquisition/reversal learning, and spatial working memory. In contrast, when all eCN are ablated embryonically, there is only a selective impairment in motor coordination behaviors. Thus, an absence of cerebellar output neurons is less disruptive than ablating *En1/2* in eCN [7]. Moreover, when the eCN are ablated embryonically, there is powerful compensation from outside the cerebellum that results in the minimum requirement for these neurons being in motor coordination and not learning and social behaviors.

## Mechanisms of Development and Regeneration in the Neonatal Cerebellum (N.S. Bayin)

The cerebellum has protracted development compared to the rest of the brain, and neurogenesis continues for up to two weeks in mice (and six months in humans). While this protracted development allows for the expansion of the cerebellum, which houses 80% of the neurons in the brain, it also makes the cerebellum susceptible to injury around birth. The postnatal development of the cerebellum is orchestrated by a series of lineage-restricted progenitors. The rhombic lip-derived granule cell progenitors give rise to the excitatory granule neurons, while the ventricular zone-derived *Nestin*-expressing progenitors (NEPs) that consist of multiple subgroups generate the inhibitory molecular layer neurons or astroglia (Bergmann glia and astrocytes) [8–11]. Interestingly, the neonatal mouse cerebellum exhibits high regenerative potential and can recover from losing Purkinje cells [12] or granule cell progenitors (GCPs) [8, 13, 14] at birth via distinct mechanisms. For example, when the GCPs are ablated at birth, a normally *Hopx* + gliogenic-NEP that resides in the Purkinje cell layer is able to undergo adaptive reprogramming – proliferate, switch to neurogenic fate, migrate to the site of injury and differentiate – giving rise to new GCPs which in turn ensure development proceeds normally [8, 13]. scRNA-seq experiments identified that adaptive reprogramming is orchestrated by *Ascl1*, a basic HLH transcription factor that is normally involved in inhibitory neuron production in the cerebellum [8]. These results highlight that context-dependent transcriptional networks promote distinct lineage outcomes during development and regeneration. The unique lineage plasticity of cerebellar NEPs provides us with a powerful model system to study the molecular mechanisms of lineage decisions and plasticity in the brain during development and regeneration. Using scRNA-seq and scATAC-seq on freshly isolated neonatal NEPs from control and injured mice, the Bayin laboratory investigates the gene regulatory networks that drive lineage decisions and tests candidate transcriptional mechanisms using innovative in vitro primary NEP cultures from neonatal mouse and fetal human cerebellar tissue.

## Cerebellar Development from the Perspective of *Pax6* and Gene Regulation (J. Yeung, D. Goldowitz)

Yeung, Goldowitz and colleagues have focused on *Pax6* as a key to gene regulatory networks (GRNs) that underpin the neuronal organization and diversity of the cerebellum’s glutamatergic constituency. Using a multi-omic approach to study normal and *Pax6* mutant early cerebellar development, they focused on three embryonic timepoints – days 13.5, 15.5, and 18.5 - using FACS-sorted *Atoh1-EGFP* + cerebella. The scRNAseq results at E13.5 and E18.5 point to discrete, early, molecular subdomains of cerebellar nuclear neurons characterized by *Pou3f1*, *Olig2* and *Tbr1*; and to an anterior-to-posterior parcellation of external granule layer cells relative to *Ostf1*, *Tlx3*, and *Otx2* with the downregulation of the first two in favor of an upregulation of *Otx2* in the mutant; with downregulation of *Neurod1*, a marker of GC differentiation.

Chromatin profiling utilizing CUT&Tag technology identified PAX6 binding sites that differ at each time point, highlighting developmental progression. Out of a total of 10,702 unique binding regions, 1180, 2700 and 2374 unique binding regions were identified in the E13, E15 and E18 glutamatergic lineages, respectively. At E13.5, 1139 genes with transcription start sites (TSSs) proximal to PAX6 binding signal peaks were differentially expressed (DE) between *Pax6* mutant and wild type glutamatergic neurons. Over 50% of these *Pax6* bound DE genes are found in the *Tbr1* + cerebellar nuclear neurons, for example *Tbr1*, *Eomes*, *Neurod1*, *Olig2* and *Lhx9* were direct PAX6 targets in this cell type that show perturbed expression in the lack of *Pax6*. These transcription factors (TFs) constitute potential regulatory targets of PAX6 to inform perturbation modeling.

microRNA/mRNA analyses of E18.5 cerebella identified a disproportionate number of upregulated miRNAs in the *Pax6*-null cerebellum. Furthermore, a surprisingly large set of novel and positively correlated miRNAs and targeted mRNAs were identified, which was unexpected. The authors are modeling the development of glutamatergic cell types through in silico GRNs. Using scRNA-seq and scATAC-seq data to model gene expression and chromatin accessibility, respectively, the GRNs will be perturbed in CellOracle [15] and SCENIC+ [16] to simulate *Pax6* knockouts and the in silico findings will be compared with *Pax6*-null scRNA-seq and PAX6 CUT&Tag-seq data to evaluate efficacy and concordance. This pipeline provides the foundation for studying other genes and cells of interest through an inference-based embryonic cerebellar development model.

## Cerebellar Granular Cell Progenitors Exit their Germinative Niche Via BarH-like1 Activity Mediated Partly by Inhibition of T-Cell Factor (B.C. Durand)

Cerebellar granule cell progenitors (GCP) originate from the upper rhombic lip (URL), a germinative niche whose developmental defects produce human diseases. T-Cell Factors (TCF) responsiveness and Notch dependence are hallmarks of self-renewal in neural stem cells. TCF activity together with transcripts coding for proneural genes repressors *hairy* and *enhancer of split* (*hes/hey*), are detected in the URL. However, their functions and regulatory modes are unknown. The Durand group established amphibian as a pertinent model to study vertebrate URL development. Amphibians long-lived URL is Tcf active, while the External Granular layer (EGL) is non-proliferative and expresses *hes4/5* genes [17]. Using functional and transcriptomic approaches, they show that Tcf activity is necessary for URL emergence and maintenance. They establish that the transcription factor *Barhl1* controls GCP exit from the URL, acting partly through direct Tcf inhibition. Identification of BARHL1 target genes argues that besides Tcf, BARHL1 inhibits transcription of *hes5* genes independently of Notch signaling. Observations in amniotes suggest a conserved role of Barhl in maintenance of the URL/EGL via coregulation of TCF and hes/hey genes.

## A human-centric View of Cerebellar Development (P. Haldipur, K.J. Millen)

Mice have been the predominant model of human cerebellar development, function, and dysfunction, but mice are not human. Haldipur and Millen now show that sequences of ventricular GABAergic and rhombic-lip glutamatergic neurogenesis are conserved from human to mice, yet all progenitor niches in humans are more complex, with extended temporal dynamics. GABAergic neurogenesis occurs very early in humans, with cerebellar nuclei neurons born prior to Carnegie Stage 21 (46 post-conception days). Human Purkinje cells are subsequently generated across a short 2-week period, prior to 8 post-conception weeks (pcw), in two waves. Initial Purkinje cells are generated from the human sub-ventricular zone (not seen in other species), followed by another wave of neurogenesis from the ventricular zone. Purkinje cells do not develop considerable dendritic complexity until several weeks later (14 pcw), when the earliest differentiated granule neurons settle in the inner granule layer. The fetal (> 8pcw) rhombic lip also develops molecular and morphological complexity not seen in other species and persists until birth [18]. The temporal and morphological expansion of the human rhombic lip has enabled us to identify progenitors and cell states likely to be extremely transitory during the rapid 5-day lifespan of the mouse rhombic lip [19]. Discovery of species differences has shifted several of prior mouse-centric paradigms of the origins of human cerebellar malformations and tumors [20–22]. By integrating interdisciplinary data from both human and mouse studies, they aim to synergistically advance our understanding of cerebellar development, function, and dysfunction, ultimately enhancing the potential for translational breakthroughs.

## Cellular Development of the Mammalian Cerebellum Through an Evolutionary Lens (M. Sepp)

The cerebellum expanded alongside the neocortex during human evolution and is increasingly recognised for its role in cognition. Despite significant advances in understanding the molecular mechanisms of neocortical evolution, those governing changes in the cerebellum remain less well characterized. To bridge this gap, Sepp and colleagues compared the developmental programs responsible for generating cellular diversity in the cerebellum across mammals [23, 24]. To this end, they generated single-nucleus gene expression and chromatin accessibility data covering cerebellum development in six species (human, bonobo, macaque, marmoset, mouse, opossum) that altogether result in a dataset of more than 700,000 single-nucleus profiles [25].

Based on these data, they established a consensus classification of the cellular diversity in the mammalian cerebellum and found largely conserved developmental dynamics of cell-type generation, except for Purkinje cells. In early fetal development, the relative abundance of Purkinje cells in humans is nearly twice that in mice or opossums, with the increase disproportionately affecting the subtypes that are born first during development [24]. They suggest that this change in Purkinje cell dynamics and patterning in humans may be related to the unique presence of subventricular progenitors in the human cerebellum, which may act as an additional source for Purkinje cell production.

Gene expression analyses revealed that cerebellar cell type-defining programs have been preserved for at least 160 million years of mammalian evolution. Consistently, Sepp and colleagues were able to identify key gene sets that contribute to the ancestral transcriptional programs shaping cell fate specification in the cerebellum. However, they also observed widespread gene repurposing at the cell-type level, identifying numerous genes that gained or lost expression during evolution. A subset of these genes is specifically recruited to the transcriptomes of subpopulations of human neural progenitor cells, potentially underlying their capacity for sustained proliferation in the cerebellar subventricular zone.

In their ongoing studies, they are using the chromatin accessibility data to infer gene regulatory networks, to decode the sequence grammar of cis-regulatory elements used in cerebellar cell types, and to identify the elements driving interspecies expression differences. Altogether, their work unveils shared and lineage-specific programs governing cerebellum development, and expands our understanding of mammalian brain evolution.

## Evolution, Development, and Connectivity of the Cerebellar Nuclei (J.M. Kebschull)

The cerebellar nuclei vary in number across vertebrate lineages, with three bilaterally symmetric nuclei in mammals, two in reptiles and birds, and one in amphibians and cartilaginous fishes. No cerebellar nuclei have been reported in jawless vertebrates [26]. This arrangement suggests the evolution of the cerebellar nuclei from a single ancestral brain region. Based on transcriptomic evidence from amniotes, Justus Kebschull and colleagues recently proposed that new cerebellar nuclei form by a process of duplication and divergence of an archetypal nucleus composed from a deeply conserved cell type set [3]. The Kebschull lab is now extending these findings to define the molecular makeup of the ancestral cerebellar nucleus by spatial sequencing in amphibians and lungfish and by investigating cerebellar nuclei development. They reported interim results of these efforts. To understand how newly formed nuclei are integrated into brain-wide circuits, the lab relies on barcoded connectomics techniques, including MAPseq [27, 28] and BRICseq [29], to map neuronal connectivity at single-cell resolution and with transcriptomic annotation. However, high resolution connectivity mapping tools are largely absent in non-mammalian systems. The Kebschull lab presented new technologies to overcome this hurdle and map connectivity at single-cell resolution across vertebrates ranging from amphibians to monkeys. Taken together, this work will illuminate the evolutionary and developmental origins of the cerebellar nuclei.

## FOXP Genes Regulate Purkinje Cell Diversity in Cerebellar Development and Evolution (J.Y.H. Li)

Mammalian cerebellar development is thought to be influenced by distinct Purkinje cell (PC) subtypes. However, the degree of PC heterogeneity and the molecular drivers of this diversity have remained unclear, hindering efforts to manipulate PC diversification and assess its role in cerebellar development. Recently, the Li group identified 11 PC subtypes in the embryonic mouse cerebellum through single-cell RNA sequencing [30]. Using a novel unsupervised method, they mapped these subtypes in three-dimensional space, revealing discrete PC subtypes predictive of adult cerebellar organization, including longitudinal stripes and lobules. These subtypes exhibit unique combinations of *Foxp1*, *Foxp2*, and *Foxp4* expression. Deletion of *Foxp2* and *Foxp1* disrupts PC diversification, leading to altered cerebellar patterning, including the loss of a specific *Foxp1*-expressing subtype and the cerebellar hemisphere. The *Foxp1*-expressing PC subtype is much more abundant in the fetal human cerebellum than in mice, but rare in the chick cerebellum, correlating with cerebellar hemisphere size in these species. This highlights the significance of *Foxp1*-expressing PCs in cerebellar hemisphere development and evolution. Therefore, their study identifies Foxp genes as key regulators of PC diversity, providing new insights into the developmental and evolutionary underpinnings of the cerebellum.

## Diving into Cerebellar Astrocyte Heterogeneity and its Development (V. Cerrato and G. Turrini)

A key question in understanding cerebellar development, function, and disease is the role of astrocytes in these processes [31]. Yet a deeper understanding of their heterogeneity is needed, guided by the hypothesis that different astrocyte subtypes may differentially contribute to cerebellar functions. In the cerebellum, with multiple neuronal subtypes [3, 24, 32], the study of astrocyte heterogeneity holds an even higher significance.

In her presentation, Dr. Valentina Cerrato showed unpublished findings on the diversity of mouse cerebellar astrocytes and its development. Traditionally, cerebellar astrocytes have been classified based on morphology and layer distribution, into four types: Bergmann glia (BG), granular layer astrocytes (GLA), cerebellar nuclei astrocytes (CNA), and white matter astrocytes (WMA) [33]. As further explored in Giacomo Turrini’s poster, by integrating new sc/snRNAseq datasets with publicly available ones [24, 32, 34], a comprehensive characterization of the full repertoire of cerebellar astrocyte types was achieved, reaching an unprecedented resolution on their heterogeneity. These analyses confirmed that the known astrocyte types exhibit distinct molecular profiles and uncovered previously unknown subtypes.

Overall, BG and the other “non-BG” astrocytes exhibited the most pronounced differences in their transcriptomes, supporting distinct functional specializations. Gene Ontology (GO) analysis confirmed that BG were more specialized in maintaining tissue architecture and regulating synaptic transmission between PCs and their inputs [35]. In contrast, novel potential functions emerged for the other astrocytes, namely interaction with blood vessels, regulation of blood supply, metabolic support to neurons, and neuroprotection.

Distinct, spatially-segregated BG subpopulations were identified by exploiting published spatial transcriptomics datasets [18, Mouse Brain Serial Sect. 1 (Sagittal-Posterior), Spatial Gene Expression Dataset by Space Ranger 1.1.0]. These results sparked a lively discussion on the potential functional relationship between BG subtypes and spatially segregated PC subtypes [24, 32]. This intriguing possibility warrants further investigation to elucidate the functional implications of BG heterogeneity in the cerebellum.

Two other subtypes drew particular interest due to their peculiar profiles. One displayed a distinctive signature reminiscent of recently identified specialized astrocytes mediating glutamatergic gliotransmission in the hippocampus [36]. Another exhibited an overall immature profile, with high expression of genes typical of neural progenitors and stem cells.

Dr. Cerrato subsequently focused on the developmental origins of this diversity. By applying a multimodal computational approach to their newly generated postnatal multi-stage sc/snRNAseq dataset, she could reconstruct the maturation trajectories of most of the subtypes mentioned above. This revealed an intriguing correspondence between previously described astroglial-like progenitor pools [9, 37, 38] and the sc/snRNAseq clusters identified at the base of distinct trajectories, allowing to uncover the molecular profiles of these progenitors.

These findings underscore the critical role of astrocyte diversity in cerebellar development and function, and open new avenues for exploring how distinct astrocyte profiles contribute to cerebellar circuitry and pathology.

## Upgrading the Physiological Relevance of Human Cerebellar Organoids (Negar Hosseini, G. Quadrato)

The lack of relevant models has constrained human cerebellar development research. Dr. Giorgia Quadrato presented a human cerebellar organoid (hCerO) system that recapitulates the cellular diversity, including human-specific rhombic lip progenitor populations, and distinct functional features of the fetal cerebellum. This system develops complex cytoarchitecture, including transient laminar organization, and demonstrates functional neuronal connections with coordinated network activity. Long-term culture allows for the maturation of Purkinje cells exhibiting molecular and electrophysiological characteristics of their in vivo counterparts in an all human system [39, 40].

This advancement is particularly significant given the unique developmental features of the human cerebellum, which exhibits distinct expansion and compartmentalization of inhibitory ventricular zone and excitatory Rhombic Lip progenitor zones, forming a human-specific subventricular zone (SVZ) [18]. Unlike mice, which lack an SVZ and have ventricular radial glial progenitors dividing only at the ventricle, humans possess mitotic progenitors in both the VZ and SVZ, with cerebellar basal progenitors serving as additional neurogenic progenitors for cerebellar development.

By integrating organoids with single-cell omics techniques and bioengineering methods, including the incorporation of synthetic molecular recorders using a novel barcode system in human induced pluripotent stem cell (hiPSC) lines and the use of multi-organoid-on-chip technologies, the Quadrato laboratory aims to achieve two main goals: (1) to foster maturation of late-born cerebellar cell type and (2) to reconstruct the developmental lineage and dynamic events of individual cells during human cerebellar neurogenesis in both health and disease.

This research will specifically investigate how human SVZ progenitors uniquely contribute to enhanced cerebellar cellular diversity and folial complexity. The integration of advanced lineage tracing techniques with single-cell transcriptomics at multiple developmental timepoints promises to reveal crucial molecular and cellular mechanisms underlying human-specific features of cerebellar development and associated neurological disorders. Ultimately, this work aims to provide new insights and tools for understanding cerebellar development and disease mechanisms, thereby contributing to foundational knowledge in the field.

## Modelling Human Cerebellar Development and Disease Using Induced Pluripotent Stem Cells (E.B.E. Becker)

Our current understanding of cerebellar development, function, and disease is largely based on animal models. However, species differences (see also “*A human-centric view of cerebellar development*”) in cerebellar development and architecture have highlighted a need for human-centric model systems to interrogate specific physiological and pathological aspects of human cerebellar development. Induced pluripotent stem cell (iPSC) technology has transformed the way human brain development and disease can be studied in vitro. In recent years, major advances have been made in the development of protocols to differentiate cerebellar neurons and organoids from human iPSCs. The Becker group has established a robust and reproducible method to differentiate dissociated cerebellar neurons and three-dimensional cerebellar organoids from human iPSCs [41–43]. These cerebellar models recapitulate early aspects of human cerebellar development including the generation of major cerebellar cell populations [43]. At the conference, Dr. Esther Becker presented two examples illustrating that cerebellar organoid models are poised to provide deeper insights into the molecular and cellular mechanisms that govern development and diseases of the human developing cerebellum. First, she presented her recent work demonstrating that consequences of aberrant sonic hedgehog signalling on neuronal specification as well as on early medulloblastoma tumorigenesis can be faithfully recapitulated in human cerebellar organoids [44]. iPSC-derived cerebellar models are increasingly used for disease modelling; however, their potential to investigate aspects of normal human cerebellar development remains underexplored. Dr. Becker shared her recent findings on using cerebellar organoids to better understand the role of Forkhead box transcription factor gene *FOXP2* in the human developing cerebellum [45]. *FOXP2* is associated with speech and language development [46] and has also recently been implicated in the early specification of Purkinje cell subtypes during mouse cerebellar development [30, 47, 48]. The Becker laboratory used CRISPR gene editing in human iPSCs to generate a fluorescent *FOXP2* reporter line and performed detailed characterisation of *FOXP2*-expressing cells in differentiated human cerebellar organoids. They showed that *FOXP2* and related FOXP family members are expressed in developing human Purkinje cells and cerebellar nuclei neurons, supporting a role for these transcription factors in defining specific cellular subtypes. Following isolation and transcriptomic analysis of the *FOXP2*-positive population of cerebellar neurons, they identified an enrichment for genes associated with neurodevelopmental disorders including autism spectrum disorder in these cells. Together, their findings highlight the value of cerebellar organoids in modelling both early neurodevelopmental processes and understanding the vulnerability of distinct cell populations to disease and thus paving the way for improved therapeutic approaches.

## Cerebellar Organoids as a Model for Pontocerebellar Hypoplasia (S. Mayer)

Pontocerebellar hypoplasia type (PCH) is a group of rare neurological disorders with onset in early infancy. Clinically, PCH is characterized by the reduced size of the cerebellum and pons as determined in magnetic resonance imaging and microcephaly developing later in childhood. The most prevalent subtype is PCH2a, which is characterized by a homozygous nucleotide variant in *TSEN54*, which encodes a tRNA splicing endonuclease (TSEN) complex subunit at the genetic level [49]. No model to date could propose a disease mechanism for PCH2a. In order to close this gap in knowledge, the Mayer Lab developed human neural organoid models of PCH2a using induced pluripotent stem cell (iPSC) lines [50]. In line with clinical observations, PCH2a cerebellar organoids were smaller than controls starting early in differentiation. Neocortical PCH2a organoids also showed reduced growth at later stages of differentiation. While no evidence for increased apoptosis in PCH2a neural organoids was found, proliferation dynamics were altered, indicating a neural differentiation deficit. Therefore, the authors suggest that PCH2a is a neurodevelopmental disorder in addition to its current classification as neurodegenerative.

The current aim of the Mayer group is to use the newly developed organoid models to elucidate the mechanisms of disease further. To do so, reproducible organoid differentiation and the use of single-cell omics to decipher altered differentiation trajectories are required. As a first step in this direction, the Mayer group has therefore compared two commercially available multiplexing techniques, 10x Genomics and Parse Biosciences [51]. They find that while both techniques generate high-quality data, the Parse workflow may be preferable for neurodevelopmental research questions, due to the lower degree of cell stress and the better capture of long genes and transcription factors. Additionally, they find variable degrees of differentiation towards a cerebellar lineage across three control iPSC lines.

## Genetic Basis and Modeling of Pontocerebellar Hypoplasia (M. Coolen, V. Cantagrel)

A confident genetic diagnosis is lacking for close to half of the patients with congenital disorders of the cerebellum. Pontocerebellar hypoplasia (PCH) are among the most severe disorders, with infantile lethality and no available treatment. Coolen, Cantagrel, and colleagues studied a cohort of PCH patients and characterized several PCH types and their molecular bases. Their work emphasizes the broad variety of mechanisms underlying PCH.

They thus identified variants in the *MINPP1* gene as the underlying cause of PCH16 [52]. This defect impacts the metabolism of inositol polyphosphates and causes an intracellular accumulation of Inositol hexakisphosphate (InsP6). InsP6 is the precursor of intracellular signaling molecules, a chelator of bivalent cations and plays a role as a structural co-factor for multiple DNA and RNA processing enzymes and protein complexes. Using patient-derived neuronal models, they identified a defect in an early stage of cortical neuron differentiation, associated with an excess of cell death. The investigation of the *Minpp1* knockout mice identified a similar differentiation defect in the cerebral cortex. In contrast, the mutant mouse does not recapitulate the severe cerebellar phenotype seen in patients, suggesting a differential sensitivity for this genetic defect.

They also discovered that mutations in *PRDM13* cause PCH17, associated with severe brainstem dysfunction [53]. PRDM13 is a transcriptional regulator containing a PR domain with predicted histone methyltransferase activity. It was previously known to be necessary for neuronal cell fate specification in the retina and spinal cord. Using single-cell expression datasets from human developing cerebellum, they showed that *PRDM13* is expressed in progenitors from the cerebellar ventricular zone. Lineage tracing in a zebrafish model demonstrated that *prdm13*-expressing progenitors generate only GABAergic neurons in the cerebellum, whereas these progenitors give rise to multiple neuronal types in the caudal hindbrain. Loss-of-function of *prdm13* results in a fate switch of hindbrain progenitors, mostly from inhibitory to excitatory neurons. Additionally, they showed that only some subtypes of Purkinje precursors express *prdm13* and critically rely on it for their differentiation. To further decrypt the underlying molecular mechanisms, in a human context, they are now generating hiPSC-derived cerebellar organoids. Their first data show that these organoids recapitulate well early steps of cerebellar neurogenesis and are thus an appropriate model to study cell fate specification defects underlying PCH17.

## Investigating Trisomy 21-Associated Hindbrain Developmental Alterations Using Human Organoids. (T.P. Silva)

Trisomy 21 (TS21) is the main cause of Down syndrome (DS), affecting approximately 1 in 1000 newborns worldwide. DS patients present with a significantly smaller brain, and the hindbrain is the most affected brain region. To understand the DS-associated hindbrain malformation, Silva and colleagues generated hindbrain organoids from healthy and TS21-derived induced pluripotent stem cell (iPSC) lines. They reported that DS-derived organoids failed to form an open apical lumen. RNA-seq data analysis revealed significant changes in global gene expression by day 14, with significant down-regulation of mRNA transcripts of apical and cell polarizing-related genes. In addition, extracellular matrix (ECM) components were altered in DS-derived organoids, while extrinsic sources of ECM can rescue the lumen phenotype in DS-derived organoids. This result suggests that significant alteration of ECM components in DS can impact the neuroepithelial polarization and the establishment of proliferative zones, an essential step in regulating brain size.

## Benchmarking Cerebellar Organoids to Model Autism Spectrum Disorder and *Sapiens* Evolution at high-resolution (D. Aprile, G. Testa)

Aprile and colleagues showed a transcriptomic benchmark of cerebellar organoids at multiple stages of differentiation *vis a vis* human fetal cerebellum at various postconceptional weeks, using an adaptation of the differentiation protocol from Muguruma and colleagues [54]. This approach led to the identification of a highly dynamic gene expression profile featuring major dysregulation in many genes causally associated to neurodevelopmental disorders, including autism spectrum disorder, in a time-window spanning 14 to 28 days of differentiation. To test how cerebellar organoids model such conditions, the authors probed the role of fully penetrant mutations in *CHD8*, a high-confidence neurodevelopmental disorder gene, causing autism, attention deficit and hyperactivity, and other associated traits. Major alterations in the trajectories of cortical neuronal development were previously identified by the Testa, Novarino and Arlotta teams [55, 56], but the role in cerebellar development was yet unknown. Single-cell RNA sequencing at early differentiation stages revealed a major alteration of the WNT pathway in *CHD8* mutant cerebellar organoids, with a dramatic change in the differentiation of granule cells progenitors and oligodendrocytes.

Additionally, the authors showed how cerebellar organoids can be used to probe the functional impact of the most recent evolutionary changes that differentiate us from our closest extinct relatives, the Neanderthals and the Denisovans. To test the sensitivity of cerebellar organoids to model the Sapiens-evolutionary changes underlying cognitive and behavioral traits affected in neurodevelopmental disorders, Aprile and colleagues used CRISPR/Cas9 editing to introduce a single nucleotide variant exclusive to archaic hominis into the enhancer of *CADPS2*, a gene involved in cerebellar development. *Cadsp2* is physiologically expressed in developing granule cells in mice, and animal models and genetic studies in individuals affected by autism and intellectual disability demonstrated a pathogenic role for this gene [57, 58]. Single-cell RNA sequencing comparing control with ancestralized cerebellar organoids showed how this variant alters the binding of oxygen sensing transcription factors and leads to an alteration of neurodevelopmental trajectories affecting specific cell populations.

In conclusion, these studies benchmark cerebellar organoids at high resolution, establishing their value to model neurodevelopmental disorders and the mechanistic dissection of the evolutionary divergence between modern and archaic humans at the cellular and molecular level.

## Human-specific Genes as Novel Medulloblastoma Drivers (L. Tiberi)

Over the past 6–10 million years, our ancestors diverged from those of chimpanzees and other great apes, acquiring genetic changes that progressively shaped the modern human species [59]. Whole-genome sequencing of modern humans, archaic hominins, chimpanzees, and other apes has enabled the identification of human-specific genetic changes, shedding light on their evolutionary significance. While these changes confer various advantages, they can also introduce new vulnerabilities.

Cancers affect all living animals; however, certain types, such as those occurring in the brain or bone tissue, are predominantly seen in humans and are rare in other species. This is particularly evident in pediatric tumors. Recent evolutionary changes have significantly transformed the human brain, bones, and immune system. The increased risk of childhood cancers affecting these tissues may represent an evolutionary trade-off for the benefits of a more complex brain and faster bone growth during adolescence.

In this study, the Tiberi group explored the relationship between brain evolution and cancer susceptibility, focusing on medulloblastoma, a pediatric tumor of the cerebellum. Human-specific genes (HSG) are key drivers of brain evolution and have been hypothesized to contribute to uniquely human vulnerabilities to disease; however, their role in cancer susceptibility remains poorly understood.

They investigated the involvement of HSGs in highly aggressive subtypes of medulloblastoma, specifically group 3 and group 4, which are believed to originate from a human-specific stem cell niche—the subventricular zone of the cerebellar rhombic lip.

Their findings reveal that a human-specific fusion gene marks a subpopulation of group 3/4 medulloblastoma cells characterized by molecular markers of the human-specific rhombic lip subventricular zone. Importantly, this HSG reduced cell death and promoted medulloblastoma growth when co-expressed with *cMYC* in the mouse cerebellum, whereas the corresponding Pan troglodytes protein did not induce tumors.

These results identify HSGs as markers of human-specific brain cancer cells and central players in medulloblastoma development. The discovery of the cancer-promoting activity of human-specific genes underscores their dual role: while driving evolutionary innovations, they also contribute to human disease susceptibility.

## Chromosome 17p13 Deletion in Human Cerebellar Development and Group 4 Medulloblastoma Oncogenesis (C. Schmidt)

The embryonic developing cerebellum is considered the origin of most pediatric hindbrain cancers, such as medulloblastoma [60]. The four subtypes of medulloblastoma arise from distinct cell types and during defined developmental windows of the immature cerebellum. To understand medulloblastoma etiology, it is necessary to uncover the consequences of genetic aberrations in vulnerable cell types during cerebellar development. Medulloblastoma subgroup group 4 (G4MB) frequently harbors haploinsufficiency of the chromosomal region 17p13 in about 63-75% of G4MB patients, and haploinsufficiency is associated with a worse prognosis. Previous studies have revealed several potential tumor suppressor genes located in the chromosomal region of interest, including *TP53*, that could play a role in medulloblastoma formation. However, it is still to be determined in what way heterozygous 17p13 chromosomal loss promotes tumorigenesis in cerebellar precursor cells and G4MB. Introducing large chromosomal deletions through genetic engineering is extremely difficult and has so far been unsuccessful. In order to study the role of 17p13 loss in medulloblastoma formation, Schmidt and colleagues are using patient-derived induced pluripotent stem cells (iPSCs) from Miller-Dieker (MDS) patients that harbor a heterozygous deletion of 17p13. They are currently characterizing the effect of 17q13 deletion in hindbrain development. MDS iPSCs can be successfully differentiated into neuroepithelial stem cells (NES), a neural stem cell population of the hindbrain. MDS NES cells show no aberrant levels of apoptosis or rates of proliferation compared to wildtype NES cells. They have also introduced forced expression of several potential G4MB oncogenes in the available MDS NES lines, such as *PRDM6*, *MYCN* and *OTX2*, and are currently studying their effect on tumor formation in vitro and in vivo. Furthermore, they are growing human cerebellar organoids from wildtype and MDS iPSCs which allows them to study human cerebellar development as a whole and under 17q13 deletion. Based on previous studies published on 17q13 deletion in the developing forebrain, they expect the chromosomal deletion to disrupt the equilibrium of proliferation and differentiation processes that are necessary during the development of the cerebellum. Their work will allow us to understand the consequences of chromosomal deletions for the developing cerebellum and G4MB formation.

## Functional Consequences of *ELP1* Mutations in vivo and in vitro (L.M. Kutscher)

Over 40% of the patients diagnosed with the SHH subtype of medulloblastoma have a germline mutation in a hereditary predisposition gene. Heterozygous mutations in *ELP1*, encoding the scaffolding subunit of the Elongator complex, accounts for nearly 15% of total hereditary predisposition to SHH-medulloblastoma [61]. Tumors from these patients harbor second hits in *ELP1* and in the SHH-receptor *PTCH1;* they are mutually exclusive with *TP53* mutations. To understand how mutations in *Elp1* lead to tumor formation, the Kutscher group modeled the end stage tumor phenotype in mouse, using the Cre-LoxP system. Therefore, they removed both copies of *Elp1* from the cell-of-origin for SHH-medulloblastoma, the granule cell progenitor, using *Atoh1-Cre* mouse line (*Elp1*^cKO^). Surprisingly, *Elp1*^cKO^ animals did not develop tumors, but instead developed ataxia by 8 weeks of age [62]. Co-mutations in *Ptch1* exacerbated the ataxia phenotype. *Elp1*^*cKO*^ cerebellum were smaller than sibling controls, which was already apparent by postnatal day P7. They hypothesized that these animals modeled Familial Dysautonomia, a hereditary neuropathy, with some defects in the CNS, characterized by a homozygous specific splice site mutation in *ELP1.*

To determine how loss of *Elp1* affects cerebellum size and function, they analyzed the developing cerebellum at P7 using immunohistochemistry, RNA-seq and proteomics. The Kutscher Group found that loss of *Elp1* increases cell death in the external granule layer, which leads to a decrease in mature NeuN+, Vglut1 + granule neurons. This cell death phenotype corresponds to increased expression of the cell cycle inhibitor p21.

Based on this mouse model, they hypothesized that single-copy loss of *ELP1* is key to understanding the medulloblastoma predisposition phenotype. Indeed, heterozygous mutations in murine GCPs led to a decreased expression of P21, compared with controls, suggesting a defect in DNA damage response and cell cycle control. To examine whether heterozygous mutations in *ELP1* could also lead to a dampened DNA damage response in human cells, they generated three iPSC clones with different heterozygous mutations in *ELP1*, all resulting in an early stop and reduced protein levels of ELP1. Following irradiation of these lines, they found the heterozygous *ELP1* iPSCs have a dampened DNA damage response, compared to controls, providing some rationale for the hereditary predisposition phenotype of *ELP1* carriers. The Kutscher Lab is currently investigating the underlying molecular mechanism, using induced GCPs differentiated from the iPSC lines and cerebellar organoids. This work will provide deeper molecular insights into the most common hereditary predisposition gene in SHH-medulloblastoma.

## scRNA-guided Identification of Novel Targets in Pediatric Group 3 Medulloblastoma (M.K. Evans II)

Medulloblastoma (MB) is the most common pediatric central nervous system malignancy. Group 3 (G3) medulloblastoma, commonly characterized by hyperactivation of the *MYC* oncogene, represents the deadliest subgroup of MB. Current standard treatment for G3 medulloblastoma includes surgery, conventional chemotherapy, and often intensive craniospinal radiotherapy, which can cause lifelong debilitating side effects. Despite our identification of *MYC* as a relevant prognostic marker and oncogenic driver, direct antagonism remains a challenging approach with significant focus on the identification of oncogene-dependent vulnerabilities. The identification of targeted therapeutic agents will be critical in enhancing patient survival and bypassing some of these therapy-derived aftereffects. The Evans Lab used a combination of published scRNA-seq [63] and functional genomics [64] to identify targets in specific subpopulations of tumor cells: a mitotic subpopulation characterized by nucleotide metabolism alterations (Grp3-A) and a progenitor population influenced by *MYC* hyperactivation (Grp3-B). Overlap of functional genomic screening data with key identifier genes in Grp3-A identified a functional dependence on *de novo* pyrimidine metabolism, particularly on an enzyme called CTPS1. *CTPS1* is elevated in medulloblastoma tumor tissue compared to normal brain and correlates with *MYC* expression in tumors. Using both genetic and pharmacological means, they demonstrated that targeting CTPS1 is cytostatic in G3 medulloblastoma cells, reducing cellular proliferation but not driving apoptosis. CTPS1 inhibition activated the replication stress response, leading to activation of ATM/ATR-CHK pathway. Blockade of this pathway with prexasertib, a potent CHK1/2 inhibitor, sensitized cells to CTPS1 inhibition leading to increased DNA damage and apoptosis both in vitro and in vivo [65].

They next targeted the Grp3-B subpopulation, which demonstrates high *MYC* expression and deregulation of chromatin modifications, specifically H3K27me3. Based on a previous publication demonstrating control of PRC2, the methyltransferase responsible for H3K27me3, they assessed the role of the RNA/DNA binding YBX1 in G3 medulloblastoma. They reveal that MYC regulates *YBX1* expression in both normal progenitors and tumor cells and identify a novel MYC-YBX1-PRC2 axis that may contribute to tumorigenesis. They show that targeting YBX1 targets stem-like progenitor cells in G3 medulloblastoma reducing self-renewal, proliferation, and tumor growth in vivo. They are currently investigating the underlying molecular mechanisms for YBX1 in regulating these processes as well as the response to current standard treatments. This work will provide a deeper understanding of the molecular insights of YBX1 in oncogenesis and lay the foundation for the continued development of novel therapeutic strategies for *MYC*-driven medulloblastoma by harnessing their discovery of a MYC-YBX1-PRC2 axis.

## Common Themes across the Conference

The talks highlighted recent progress in understanding developmental mechanisms at the epigenetic, transcriptional, and cellular levels and underscored how understudied this research topic is. Many open questions and unknowns remain, especially compared to related fields such as neocortical development. The evolutionary talks demonstrated that diverse model organisms including *Xenopus leavis*, *Danio rerio* (zebrafish), the mouse and the marsupial opossum are helpful to understand structure-function relationships and behavioral outcomes. The contributions of human in vitro models including cerebellar organoids are promising and a field of growing interest; however, there is still a need to standardize and optimize protocols and readouts, including quality control standards. In conclusion, the discussions crystallized the growing notion that the cerebellum is critical for many brain functions beyond motor control, including higher-order cognitive functions and speech. Malformations contribute to a range of neurological and psychiatric disorders, for example ASD. We expect such an exciting and growing field of research to generate major breakthroughs in the coming years.

## Conclusion and Future Directions

The “*Cerebellar development and disease at single-cell resolution*” conference showcased recent progress in the field, as highlighted above. Because of the focused and friendly atmosphere of the Cerebellar Development community, most researchers shared unpublished data in their talks and in these summaries. Furthermore, discussions in the coffee breaks and conference dinner focused on sharing tips and tricks of different cerebellar organoid differentiation protocols and animal modeling expertise. This openness created a strong sense of community, which undoubtedly will be crucial to further develop this field internationally in the next few years. The feedback was overwhelmingly positive, and participants were grateful for a meeting where every talk was relevant to one’s own cerebellar development research. While a follow-up conference would be desirable, the discussion revealed that the current funding landscape may hinder such a follow-up. Instead, it was suggested to organize cerebellar development sessions at major neuroscience conferences, such as the annual meeting of the Society for Neuroscience (SfN) or the Forum of the Federation of European Neuroscience Societies (FENS). Our community aims to stay connected through different channels, including through the communication tool Slack and a recurring Zoom web series. Please feel free to reach out to the corresponding authors to join these initiatives and become part of this collaborative community!

## Data Availability

No datasets were generated or analysed during the current study.
